# *Mycoplasma bovis*: Mechanisms of Resistance and Trends in Antimicrobial Susceptibility

**DOI:** 10.3389/fmicb.2016.00595

**Published:** 2016-04-27

**Authors:** Inna Lysnyansky, Roger D. Ayling

**Affiliations:** ^1^Mycoplasma Unit, Division of Avian and Aquatic Diseases, Kimron Veterinary InstituteBeit Dagan, Israel; ^2^Department of Bacteriology, Animal and Plant Health AgencyAddlestone, UK

**Keywords:** *Mycoplasma bovis*, minimum inhibitory concentrations, tetracyclines, macrolides, aminoglycosides, fluoroquinolones, chloramphenicols, pleuromutilins

## Abstract

*Mycoplasma bovis* is a cell-wall-less bacterium and belongs to the class *Mollicutes*. It is the most important etiological agent of bovine mycoplasmoses in North America and Europe, causing respiratory disease, mastitis, otitis media, arthritis, and reproductive disease. Clinical disease associated with *M. bovis* is often chronic, debilitating, and poorly responsive to antimicrobial therapy, resulting in significant economic loss, the full extent of which is difficult to estimate. Until *M. bovis* vaccines are universally available, sanitary control measures and antimicrobial treatment are the only approaches that can be used in attempts to control *M. bovis* infections. However, *in vitro* studies show that many of the current *M. bovis* isolates circulating in Europe have high minimum inhibitory concentrations (MIC) for many of the commercially available antimicrobials. In this review we summarize the current MIC trends indicating the development of antimicrobial resistance in *M. bovis* as well as the known molecular mechanisms by which resistance is acquired.

## Introduction

*Mycoplasma bovis* is a cell-wall-less bacterium and belongs to the class *Mollicutes* (Razin, 1978). It is the most important etiological agent of bovine mycoplasmosis in North America and Europe, causing respiratory disease, mastitis, otitis media, arthritis, and reproductive disease (Nicholas and Ayling, 2003). Until *M. bovis* vaccines are universally available, sanitary control measures and antimicrobial treatment are the only approaches that can be used in attempts to control *M. bovis* infections. However, *M. bovis* is refractory to ß-lactams and to all antimicrobials that target the cell wall. In addition, mycoplasmas are also naturally resistant to polymyxins, sulfonamides, trimethoprim, nalidixic acid, and rifampin (Taylor-Robinson and Bebear, 1997). Antimicrobials that are mycoplasmastatic provide an opportunity for the host's immune response to develop and counteract infection, but *M. bovis* has defense mechanisms that include an ability to vary its surface proteins (Lysnyansky et al., 1996) and form biofilms (McAuliffe et al., 2006).

The potential effectiveness of antimicrobials *in vivo* can be assessed by *in vitro* susceptibility testing to determine the minimum inhibition concentration (MIC). Guidelines for *in vitro* testing of veterinary mycoplasma species have been published (Hannan, 2000). However, the data produced are subject to interpretation as there are currently no *in vitro* testing standards and interpretive breakpoints have not been determined. Standards for testing *Mollicutes* that cause clinical infections in humans *Mycoplasma pneumoniae, Mycoplasma hominis*, and *Ureaplasma urealyticum* have been published (Waites et al., 2012). However, the growth requirements of these species differ from those of *M. bovis*. In addition, the authors are not aware of specific published *M. bovis* pharmacokinetics (PK)/pharmacodynamics (PD) studies which can influence the interpretation of *in vitro* antimicrobial sensitivity.

Herein, we review *in vitro* MIC data that indicate that *M. bovis* is developing antimicrobial resistance, which is increasingly being supported by molecular evidence of genetic mutations consistent with antimicrobial resistance and by reports of treatment failure.

## Antimicrobial classes active against *M. bovis*

Control of *M. bovis* infection requires early identification and treatment with antimicrobials including the tetracyclines, macrolides and some fluoroquinolones. However, few are specifically approved for treating *M. bovis* in cattle and with the exception of the fluoroquinolones, which are mycoplasmacidal, and the aminoglycosides, which are mycoplasmacidal at high concentrations, the remaining antimicrobials are mycoplasmastatic, and generally work by inhibiting protein synthesis. The typical tetracyclines, chlortetracyline and doxycycline which are polycyclic structures of the perhydronaphthacene carboxamide inhibit protein synthesis in the ribosome by binding to the 30S ribosomal subunit and blocking an attachment of aminoacyl-tRNA to the A site (Bryskier, 2005a). Other plasmid related resistance, or efflux mechanisms present in some *Mycoplasma* species have not been described in *M. bovis*.

The macrolides which are known to be good for treating respiratory infections have been shown to have a preferential distribution *in vivo*, concentrating in diseased lungs, up to three times the concentration detected in healthy lungs (Reeve-Johnson, 1999). Macrolides are hydrophobic molecules that have a central 12-16-membered-ring lactone with few or no double bonds. They inhibit protein synthesis possibly by preventing peptidyltransferase from adding the growing peptide attached to tRNA to the next amino acid as well as inhibiting ribosomal translation. Another potential mechanism is premature dissociation of the peptidyl-tRNA from the ribosome. Macrolides can penetrate phagocytic cells thereby allowing treatment of intracellular infections. Lincosamides consist of an amino acid and a sugar connected by an amide bond. They act on the 50S subunit of the ribosome, preventing transpeptidation by inhibiting peptidyl-transferase.

The fluoroquinolones are synthetic antimicrobials and all have a pyridine-ß-carboxylic nucleus. Their activity depends on several factors: the aromatic system associated with the pyridine-ß-carboxylic acid nucleus, the substituents, and their spatial disposition. These substituents produce greater affinity for the target enzymes (DNA gyrase and topoisomerase IV) but also allow penetration of the bacterial outer membrane (Bryskier, 2005b). The activity of these antimicrobials can be affected by pH, magnesium and calcium ions.

The aminoglycosides are amino sugars and fall into two broad groups based on their chemical structures: streptomycin and its derivatives and deoxystreptamines. The ribosome is the prime target of their action, but other actions on membranes and modifications of RNA synthesis have been observed. They are considered mycoplasmacidal, but that is concentration dependent.

The phenicols are derived from dichloroacetic acid with an aromatic nucleus and an alkyl group and an aminopropanediol chain. The fluoro derivative of chloramphenicol, florfenicol, is commonly used in the veterinary field. It inhibits protein synthesis by binding to the 50S ribosomal subunit inhibiting the peptidation reaction and the translation of bacterial mRNA.

Pleuromutilins, such as tiamulin and valnemulin are semisynthetic derivatives that have a tricyclic diterpenoid structure with different side chains. They have been used in veterinary medicine; they bind to the peptidyl transferase component of the 50S ribosomal subunit to inhibit protein synthesis (Long et al., 2006). Tiamulin was the first pleuromutilin compound to be approved for veterinary use in 1979, followed by valnemulin in 1999 (Novak and Shlaes, 2010).

## *M. bovis in vitro* susceptibility profiles and trends

*M. bovis* treatment failures are increasingly being associated with antimicrobial resistance. However, different research groups report variations in the proportion of *M. bovis* isolates with decreased susceptibility. This may be related to geographical origin, year of isolation, type of livestock production system, clinical presentation, or site of isolation of the strains tested. However, this may also indicate differences among the countries in regulatory practices for use of antimicrobials. Not all studies included epidemiology or typing of *M. bovis* isolates, but those which did, reported that in some countries the acquisition of resistance to antimicrobials in *M. bovis* was attributed to the emergence and spread of a single clone (Becker et al., 2015) while in other the genetic heterogeneity of *M. bovis* isolates with decreased susceptibility was identified (Lysnyansky et al., 2009).

*M. bovis* susceptibility testing has a role in epidemiological monitoring of acquired resistance and can guide the veterinarian in the selection of the most likely effective antimicrobial treatment, but published MIC data requires some interpretation, as there is no agreed standard method or controls for susceptibility testing of *M. bovis*. Although guidelines were produced by Hannan (2000), variations in the growth and approaches to determining the endpoint by different laboratories are reported and make it hard to compare results from different laboratories. However, these differences do not detract from the trends observed, which are now being supported by genetic evidence of resistance correlated with high MIC values (Lysnyansky et al., 2009; Sato et al., 2013; Lerner et al., 2014; Amram et al., 2015; Khalil et al., 2015). Nevertheless, standardized methods, controls and breakpoint interpretations would be highly beneficial for future epidemiology assessment of antimicrobial sensitivities and treatment guidance. In Table 1 we present the susceptibility profiles (ranges of MICs, MIC_50_, and MIC_90_), obtained by microdilution and agar dilution methods, of *M. bovis* isolated in different countries since 1990. Both the MIC_50_ and MIC_90_ may indicate shifts in the susceptibility of bacterial populations to antimicrobials since MIC_50_ values show the general potency of an antimicrobial against a specific type of bacteria, while the MIC_90_ values indicates the potential and first stages of specific bacteria developing resistance to an antimicrobial.

**Table 1:** **Minimal inhibitory concentration ranges (μg/ml) and MIC_**50**_ and MIC_**90**_ ranges for various antimicrobials against ***M. bovis***^**a**^**.

**Antimicrobials**	**MIC^b^ Range**	**MIC_50_ Range**	**MIC_90_ Range**
***Tetracyclines***			
Tetracycline	< 0.03–>64	1–8	4–64
Oxytetracycline	0.05–128	2–128	4–128
Chlortetracycline	0.25–256	2–128	2–256
Doxycycline	≤ 0.03–1	0.5	1
***MLSK Group***			
Erythromycin	4–>512	>3.2–512	32–512
Tylosin	0.2–>256	1–>128	6.25–>128
Tilmicosin	≤ 0.5–>512	2–512	4–>512
Gamithromycin	128–>128	128	128
Tildipirosin	128–>128	128	128
Tulathromycin	≤ 0.063–>128	0.25–128	8–>64
Kitasamycin	1–128	4	64
Josamycin	0.78–12.5	1.56	6.25
Spiramycin	0.39–>100	3.12	25
Lincomycin	0.06–>256	0.78–>64	3.12–>64
Clindamycin	0.03–>256	0.5–8	>32
Pirlimycin	< 0.125–0.5	0.25	0.5
***Pleuromutilins***			
Tiamulin	< 0.03–2	0.06–0.2	0.78–1
Valnemulin	< 0.03	< 0.03	< 0.03
***Fluoroquinolones***			
Enrofloxacin	0.025–>32	0.12–1	0.12–32
Danofloxacin	0.078–32	0.156–0.5	0.312–8
Marbofloxacin	0.25–>32	0.625–1.25	0.625–32
Flumequine	16–128	64	128
Ciprofloxacin	0.125–1	0.5	1
***Aminoglycosides***			
Gentamycin	2–>64	8	32
Kanamycin	8–>128	32–64	64–>128
***Aminocyclitol***			
Spectinomycin	0.03–>256	1–>256	2–>256
***Chloramphenicols/Phenicols***			
Chloramphenicol	0.25–>32	2–8	2–32
Thiamphenicol	3.12–>512	6.25–>512	12.5–>512
Florfenicol	0.06–64	1–8	2–32

a *The data are compiled from multiple published studies (Ayling et al., 2000; Hirose et al., 2003; Thomas et al., 2003; Rosenbusch et al., 2005; Godinho, 2008; Gerchman et al., 2009; Uemura et al., 2010; Soehnlen et al., 2011; Kroemer et al., 2012; Hendrick et al., 2013; Ayling et al., 2014; Gautier-Bouchardon et al., 2014; Kawai et al., 2014; Sulyok et al., 2014; Kong et al., 2016) in which different methodologies (microbroth and agar dilution, but not E-test) and different antimicrobial concentrations were used (adapted from Bebear and Kempf, 2005; Waites et al., 2014)*.

b *Only MIC data obtained for M. bovis isolated after 1990 were included*.

### Tetracyclines

*In vitro* susceptibility data from all over the world reveal heterogeneity in the susceptibility pattern to tetracyclines with MIC_50_ values for oxytetracycline ranging from 2 to < 8 μg/ml for isolates from Belgium, North America and Israel (Thomas et al., 2003; Rosenbusch et al., 2005; Gerchman et al., 2009; Soehnlen et al., 2011; Hendrick et al., 2013); while the MIC_50_ of isolates from the UK, the Netherlands, France, Hungry and Japan (Ayling et al., 2000, 2014; Hirose et al., 2003; Uemura et al., 2010; Gautier-Bouchardon et al., 2014; Kawai et al., 2014; Sulyok et al., 2014) was reported to be ≥8 μg/ml. Notably, when susceptibility to tetracyclines was tested in two cohorts of respiratory *M. bovis* isolates isolated in the UK in 2004 and 2009, a significant increase in the MIC_50_ from 1 to 32 μg/ml was identified (Ayling et al., 2014). In contrast, in France, the group of archived isolates (1978–1979) already demonstrated high MIC_50_ to oxytetracycline (32 μg/ml), which increased to ≥32 μg/ml in recent isolates (2010–2012) (Gautier-Bouchardon et al., 2014). The authors suggested that less susceptible *M. bovis* isolates had developed by 1978–1979.

### Macrolides and lincosamides

Acquired resistance to macrolides in *M. bovis* is a widely known phenomenon and isolates with high MICs are frequently identified (Table 1). For example, MIC_50_ values of ≥32 μg/ml for tilmicosin alone or for tylosin and tilmicosin were identified in recent British and Hungarian isolates (Ayling et al., 2014; Sulyok et al., 2014) as well as in isolates from western Canada, Japan and the USA (Rosenbusch et al., 2005; Uemura et al., 2010; Hendrick et al., 2013; Kawai et al., 2014). All recent French isolates had MICs of ≥64 μg/ml for tylosin and tilmicosin (Gautier-Bouchardon et al., 2014). Gerchman et al. (2009) reported marked differences in susceptibility profiles to tylosin and tilmicosin in isolates from different geographical regions. Israeli isolates were significantly more resistant to macrolides than isolates from calves imported from Hungary (2005–2007). However, recent Hungarian isolates (2010–2013) are more resistant to these antimicrobials (MIC_50_ and MIC_90_ ≥128 μg/ml) (Sulyok et al., 2014). Such data emphasize the requirement for periodic antimicrobial susceptibility testing on a regional basis.

Both archived and recent French isolates possessed high MICs for the new-generation macrolides, gamithromycin and tildipirosin. In addition, all tested French isolates and 27% of British tested isolates had MIC values of ≥128 μg/ml to tulathromycin, a relatively new semi-synthetic macrolide (Ayling et al., 2014; Gautier-Bouchardon et al., 2014). Interestingly, results of a susceptibility study comparing MICs for tulathromycin in *M. bovis* respiratory isolates isolated in Europe prior to 2002 and between 2004 and 2006 showed an increase in MIC_50_ (from 0.25 to 4 μg/ml) without a change in the MIC_90_ (>64 μg/ml) (Godinho, 2008). The authors suggested that a high MIC does not necessarily reflect a lack of clinical efficacy of tulathromycin since an isolate with an MIC >64 μg/ml was effectively treated in an experimental trial (Godinho et al., 2005; Godinho, 2008).

The MIC_50_ for lincomycin ranged from 0.78 to ≥64 μg/ml in several studies (Hirose et al., 2003; Thomas et al., 2003; Uemura et al., 2010; Ayling et al., 2014; Sulyok et al., 2014) and MIC_50_ of 0.19 μg/ml (but with MIC_90_ of >256 μg/ml; tested by *E*-test) for clindamycin were reported from Canada (Francoz et al., 2005). Interestingly, Ayling et al. (2014) reported decreased MIC_50_ values for lincomycin (from 8 to 1 μg/ml) and clindamycin (from ≥32 to 0.25 μg/ml) in *M. bovis* isolates isolated in the UK in 2004 and 2009. Low MIC_50_ and MIC_90_ of 0.25 and 0.5 μg/ml, respectively for pirlimycin (Table 1) were reported in one study from Japan (Kawai et al., 2014).

### Fluoroquinolones

In contrast to acquired resistance to macrolides which has been reported for a long time, the resistance to veterinary fluoroquinolones is a relatively new phenomenon. For example, only a single fold increase in MIC_50_ and MIC_90_ (in both cases from 0.25 to 0.5 μg/ml for enrofloxacin and danofloxacin; and from 0.5 to 1 μg/ml for marbofloxacin) was reported between 1978–1979 and 2010–2012 for French isolates (Gautier-Bouchardon et al., 2014). The same increase in the MIC_50_ was also found in UK isolates isolated between 2004–2005 and during 2007–2009. However, their MIC_90_ values differed considerably (0.5−1 vs. 8−>32 μg/ml) (Ayling et al., 2014). Relatively low enrofloxacin MIC_50_ values of 0.12 to 0.5 μg/ml were reported in North America, Japan and some European countries (Hirose et al., 2003; Thomas et al., 2003; Rosenbusch et al., 2005; Uemura et al., 2010; Soehnlen et al., 2011; Hendrick et al., 2013; Gautier-Bouchardon et al., 2014; Kawai et al., 2014; Sulyok et al., 2014). This may reflect differences in therapeutic use of antimicrobials in different countries.

### Aminocyclitol and aminoglycosides

Several studies have demonstrated relatively low MIC_50_ and MIC_90_ values for spectinomycin (up to 8 μg/ml) (Rosenbusch et al., 2005; Uemura et al., 2010; Hendrick et al., 2013), while others reported that the difference between the MIC_50_ (1–8 μg/ml) and MIC_90_ (>32 μg/ml) was high (Ayling et al., 2000, 2014; Thomas et al., 2003; Soehnlen et al., 2011; Sulyok et al., 2014). In addition high MIC_50_ and high MIC_90_ (≥256 μg/ml) values for spectinomycin were also reported (Ayling et al., 2000, 2014; Thomas et al., 2003; Soehnlen et al., 2011; Sulyok et al., 2014). Interestingly, higher spectinomycin MIC values have been reported for isolates from milk. Rosenbusch et al. (2005) reported that 6 out of 14 USA milk isolates had MIC values >16 μg/ml while Soehnlen et al. (2011) reported a significant difference between MIC_50_ of milk (>256 μg/ml) and lung (8 μg/ml) isolates. MIC values for gentamycin ranged from 2 to >64 μg/ml (Thomas et al., 2003; Sulyok et al., 2014) and kanamycin from 8 to >128 μg/ml (Uemura et al., 2010; Kawai et al., 2014).

### Chloramphenicols

The MIC_50_ values for florfenicol ranging from 1 to 8 μg/ml were identified in North America, Europe and China (Rosenbusch et al., 2005; Soehnlen et al., 2011; Hendrick et al., 2013; Kong et al., 2016). In addition, *M. bovis* isolates from Japan gave MIC_50_ values of 6.25-8 μg/ml and 6.25->512 μg/ml to chloramphenicol and thiamphenicol, respectively (Hirose et al., 2003; Uemura et al., 2010). Due to the potential toxicity of phenicols and chloramphenicols (not florfenicol), their use is now illegal in food animals in many countries (Papich and Riviere, 2013).

### Pleuromutilins

The few MIC studies on tiamulin and valnemulin showed low MIC values for both antimicrobials (Table 1; Thomas et al., 2003; Gautier-Bouchardon et al., 2014). The efficacy of valnemulin against *M. bovis* infection was also demonstrated *in vivo* (Stipkovits et al., 2001, 2005).

## Molecular mechanisms of *in vivo* acquired resistance by *M. bovis*

In general, until now, the only mechanisms of acquired *in vivo* resistance described in *Mollicutes* are target modification and ribosome protection by the *tet*(M) determinant (Bebear and Kempf, 2005).

### Tetracyclines

The genetic background for decreased tetracycline susceptibility has been elucidated in greater detail in mycoplasmas of human origin than in *M. bovis* (reviewed by Waites et al., 2014). Ribosomal protection by *tet*(M) determinants in *M. hominis* and *Ureaplasma* spp. tetracycline-resistant isolates was identified (Roberts et al., 1985, 1986) while target modification with point mutation(s) in the 16S rRNA genes was described for *in vitro* obtained mutants of *M. pneumoniae* with MICs of ≤ 2 μg/ml (Degrange et al., 2008). Recent correlation between decreased susceptibility to tetracycline hydrochloride in *M. bovis* isolates and mutations identified in the primary binding pocket for tetracycline (Tet-1 site) located in 16S rRNA-encoding genes (*rrs3* and *rrs4* alleles) was shown (Amram et al., 2015). An increase in MICs to tetracycline (MICs ≥2 μg/ml) was correlated with the number of mutated nucleotide positions within the Tet-1 site. Indeed, decreased susceptibilities to tetracycline were associated with mutations present at two (A965 and A967; *E. coli* numbering) or three positions (A965, A967, and G1058) in the two *rrs* alleles. No *tet*(M), *tet*(O), or *tet*(L) determinants were identified in any of the 70 *M. bovis* isolates tested. Notably, no *M. bovis* isolates with high MICs (≥32 μg/ml) to tetracycline were available and tested in this study.

### Macrolides and lincosamides

Macrolide resistance in mollicutes, which have 1–3 ribosomal operons, is caused by point mutations of the macrolide-binding site located in the 23S rRNA genes or in the genes encoding ribosomal proteins L4 and L22 (reviewed by Waites et al., 2014). Recently, *M. bovis* decreased susceptibility to tylosin and tilmicosin was attributed to any one of the point mutations G748A, C752T, A2058G, A2059G, or A2059C (*E. coli* numbering) identified in one or both alleles of the 23S rRNAs (Lerner et al., 2014). The data revealed that a combination of mutations in two domains (II and V of 23S rRNA) was necessary to achieve higher MICs (≥128 μg/ml) to tylosin, while a single mutation in one of the domains could be sufficient to cause a decrease in susceptibility to this antimicrobial. In that study, amino acid (aa) substitution L22-Q90H (*E. coli* numbering) was also identified in 24 of 32 representative *M. bovis* isolates with different MICs, but no clear correlation between this mutation and decreased MICs to tylosin and tilmicosin was found. In addition, multiple aa substitutions were identified in the L4 protein, including at positions 185–186 (positions 64 and 65 in *E. coli*), which are adjacent to the macrolide-binding site (Lerner et al., 2014). However, the actual impact of L4 aa substitutions is hard to define since they were identified in isolates with various MICs, some of which also possessed nt substitution/s within the macrolide binding site of 23S rRNA gene/s. Clarification of the role of mutations at positions 90-L22 and 64/or 64-65-L4 in decreased macrolide susceptibility by *M. bovis* is required.

### Fluoroquinolones

The only mechanism of acquired fluoroquinolone resistance described in *Mollicutes* is alterations within the quinolone resistance-determining regions (QRDRs) of DNA gyrase subunits GyrA and GyrB and/or topoisomerase IV subunits ParC and ParE. However, an active efflux mechanism has also been demonstrated *in vitro* (Raherison et al., 2002). Amino acid substitution in the QRDR of GyrA (Ser83Phe) alone or with concurrent mutation Asn84Asp in ParC was found in *M. bovis* isolates with MICs to enrofloxacin ranging from 0.32–1.25 to 2.5–5 μg/ml, respectively (Lysnyansky et al., 2009). In another study, mutation Ser83Leu in GyrA was identified in 32 isolates with MICs of 0.25–2 μg/ml to enrofloxacin, orbifloxacin and danofloxacin; mutations in Ser83Leu of GyrA and Ser81Pro of ParC were present in three isolates (4–16 μg/ml) while mutations Ser83Phe in GyrA and Ser80Ile in ParC were found in four isolates (8–16 μg/ml) (Sato et al., 2013). The mutations Ser83Phe in GyrA and Ser80Ile in ParC were also identified in a Chinese *M. bovis* isolate with MICs ≥16 μg/ml to ciprofloxacin, levofloxacin, lomefloxacin and norfloxacin (Mustafa et al., 2013). In addition, Sato et al. (2013) showed that laboratory-derived fluoroquinolone-resistant mutants selected from two isolates with Ser83Leu in GyrA possessed an aa substitution Ser80Ile or Ser81Tyr in ParC. Results of both studies (Lysnyansky et al., 2009; Sato et al., 2013) suggest a cumulative effect of the mutations in GyrA and ParC on fluoroquinolone resistance. Previously, *in vivo* acquired resistance by veterinary mycoplasmas to the fluoroquinolones has been attributed to aa changes at positions 80 or 84 in the ParC as well as positions 83 in the GyrA (Hirose et al., 2004; Le Carrou et al., 2006; Vicca et al., 2007).

### Aminoglycosides

No enzymes responsible for chemical modification of aminoglycosides have been identified in the *M. bovis* genome. However, decreased susceptibility to streptomycin in *M. bovis* was associated with nt T at position 912 (*E. coli* numbering) in the 16S rRNA genes (Konigsson et al., 2002), a position converting to resistance in *E. coli* (Frattali et al., 1990).

In an aminocyclitol antimicrobials, spectinomycin, isolates with high MIC (>512 μg/ml) were found to exhibit a C to A transition at position 1192 (*E. coli* numbering) either in *rrs3* or in both *rrs* alleles (C. Schnee et al., abstract, IOM 2014, p. 60) as has been previously shown for spectinomycin-resistant *E. coli* (Sigmund et al., 1984).

### Chloramphenicols and pleuromutilins

To the best of our knowledge, no investigations of the phenicols /chloramphenicols and pleuromutilins resistance mechanisms for *M. bovis* have been published.

## Summary

Control of *M. bovis* infections in cattle is inherently difficult. The resulting clinical signs and disease are increasingly recognized as having a significant adverse impact on animal welfare and the economy of cattle farming around the world. Other than sanitary preventative measures, treatment with antimicrobials is the only approach for disease treatment. However, as emphasized herein, the number of potentially effective antimicrobials is limited as *M. bovis* lacks a cell wall. Among the few antimicrobials licensed for treatment of *M. bovis*, there is increasing evidence for resistance. Based on MIC levels and genetic analysis, antimicrobial resistance by *M. bovis* to the tetracyclines, macrolides, lincosamides, aminoglycosides, chloramphenicols, and fluoroquinolones has been reported and appears to be increasing. The mechanisms of *M. bovis* antimicrobial resistance have largely been based on genetic point mutations; few studies have examined efflux mechanisms and no plasmids have so far been detected in *M. bovis*.

This review highlights the necessity to agree a standardized method and controls for animal mycoplasma antimicrobial susceptibility testing. In addition breakpoints should be determined and the use of molecular resistance markers could be used to define them.

## Author contributions

IL, RA designed the study and analyzed and interpreted data. IL, RA drafted the manuscript. All authors read and approved the final version of the manuscript.

### Conflict of interest statement

The authors declare that the research was conducted in the absence of any commercial or financial relationships that could be construed as a potential conflict of interest.
